# The Distribution of *Wolbachia* in *Cubitermes* (Termitidae, Termitinae) Castes and Colonies: A Modelling Approach

**DOI:** 10.1371/journal.pone.0116070

**Published:** 2015-02-11

**Authors:** Virginie Roy, Marc Girondot, Myriam Harry

**Affiliations:** 1 iEES—Institut d’écologie et des sciences de l’environnement de Paris, Département SOLéO, Université Paris-Est Créteil, Faculté des Sciences et Technologie, 61 avenue du Général de Gaulle, 94010 Créteil cedex, France; 2 Laboratoire d’Ecologie, Systématique et Evolution, Département d’Ecologie des Populations et des Communautés, Université Paris-Sud 11, Bâtiment 362, 91405 Orsay Cedex, France; 3 Laboratoire Evolution, Génomes et Spéciation, UPR 9034 CNRS, UR 072 IRD, Université Paris Sud-11, avenue de la Terrasse, Bâtiment 13, 91198 Gif sur Yvette, France/ UFR de Sciences, Université Paris-Sud 11, 91400 Orsay, France; International Atomic Energy Agency, AUSTRIA

## Abstract

*Wolbachia* are endosymbiotic bacteria of arthropods and nematodes that are able to manipulate host reproduction. Although vertically transmitted via the cytoplasm in eggs, horizontal transmission of *Wolbachia* among and within arthropod species has been shown to be common. Eusocial insects represent interesting models for studying *Wolbachia* transmission due to colonial organization and close interaction between nestmates. Here we conducted a detailed screening of *Wolbachia* infection for 15 colonies of the very common soil-feeding termites *Cubitermes* spp. *affinis subarquatus* (Termitidae, Termitinae) that consist of four distinct phylogenetic species in the Lopé forest Reserve, Gabon. Infection tests showed that 50% of the individuals were *Wolbachia* positive (N = 555) with 90% of reproductives and 48% of offspring infected. White soldiers, which are transitional stages preceding mature soldiers, had a significantly higher mean infection rate (74%) than the other castes and stages (63%, 33% and 39% for larvae, workers and mature soldiers, respectively). We used a maximum likelihood method and Akaike’s Information Criterion in order to explain the non-expected high rate of *Wolbachia* infection in white soldiers. The best model included a species effect for the stochastic loss of *Wolbachia* and a caste effect for the rate of gain. After fitting, the best model selected was for a species-specific rate of loss with a null rate of new gain for larvae, workers and soldiers and a probability of 0.72 whatever the species, that a white soldier becomes newly contaminated during that stage. The mean expected infection rate in white soldiers without a new gain was estimated to 17% instead of the 74% observed. Here we discuss the possible explanations to the high infection rate observed in white soldiers.

## Introduction

*Wolbachia* is an endosymbiotic bacterium that infects a wide range of arthropod and nematode species [1–3]. This parasite has been shown to increase its transmission via manipulation of reproduction and sex ratio of arthropod hosts using different mechanisms: cytoplasmic incompatibility, parthenogenesis induction, male killing and feminization of genetic males [4–8]. *Wolbachia* infections were initially thought to be restricted to the germ-line tissue of their hosts, but results in a variety of insects and isopods demonstrated that they have a wide tissue tropism [9,10] and are detected in various somatic tissues comprising muscles, gut, head and salivary glands [5,11–13]. Although vertically transmitted via the cytoplasm in eggs (i.e. maternally inherited), the lack of congruence observed between the phylogenetic tree of *Wolbachia* and that of their hosts [3], and observations of natural interspecific and intraspecific transfer of the bacteria [14,15], suggested a degree of horizontal transmission both between and within arthropod host species.

Due to their colonial organization with permanent contact and trophic interactions between nestmates, eusocial insects are particularly interesting for studying *Wolbachia* transmission. *Wolbachia* prevalence in eusocial bees and wasps has received growing interest in recent years [16–19]. The bacterium appears to be widespread in ants with about one third of worldwide ant species being infected [2,20–22]. Studies investigating infection rates in ant populations and colonies have provided interesting lines of research concerning dynamics of *Wolbachia* [20,23–30]. In termite species, *Wolbachia* was described with a prevalence of 27% [31] and a great strain diversity was identified in this group [31–35]. However, to our knowledge no information is available concerning the infection status within termite colonies.

**Fig 1:**
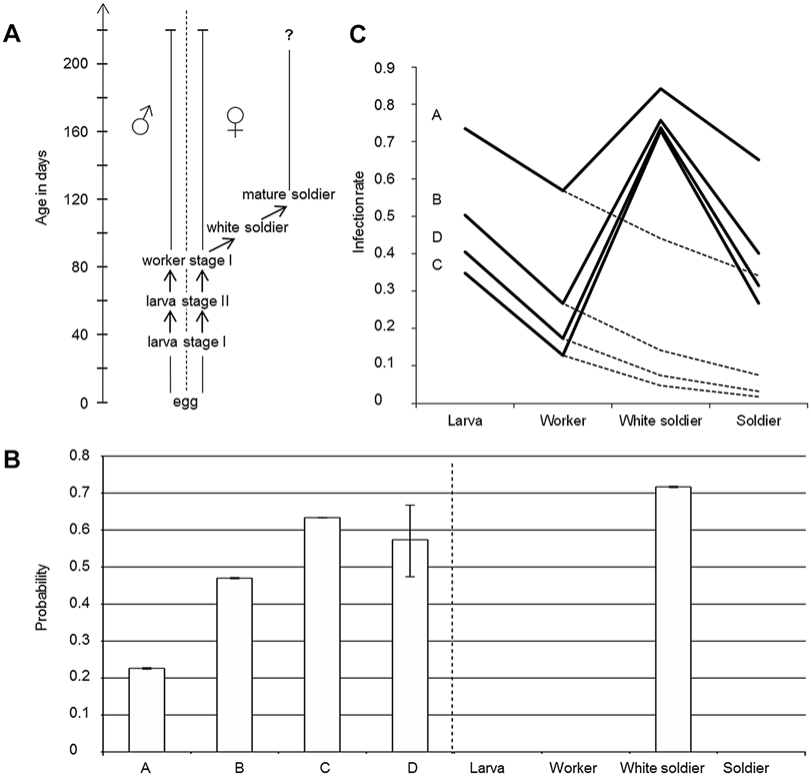
a) Development of non-sexual lines in the genus *Cubitermes* synthetized from various reports about *Cubitermes* species life cycle [36,79–81]. Larval stages comprise males and females, which both differentiate into a single stage of male and female workers (Worker stage I). A subset of the female workers further differentiates into pre-soldiers (i.e. white soldiers, transitional stage preceding mature soldiers), b) Left: fitted parameters for the selected model: probability of stochastic loss of *Wolbachia* between two stages according to the species and right: probability of gain of *Wolbachia* during stages. c) Observed (bold lines) and expected under the model (dotted lines) infection rates for the four species *Cubitermes* sp. A, B, C and D and stages/castes.

Termites (Blattodea: Termitoidae), albeit also eusocial insects, differ in many ways from eusocial Hymenoptera. In particular, most termite species possess a differentiated soldier caste and numerous molts throughout the lifecycle of individuals (i.e. termites are heterometabolous insects). Due to their non-cryptic colonial organization in epigeous nests, termites of the family Termitidae, subfamily Termitinae, are ideal models for studying *Wolbachia* distribution and exchanges. Colonies are headed by a royal pair, called the primary king and queen. Larval stages differentiate into two lines, a sexual line which produces nymphs i.e. future winged imagoes (during a short seasonal period), and a sterile line which produces true workers. A subset of the workers (males and/or females depending on the species, typically females for *Cubitermes* species) further differentiate into pre-soldiers (white soldiers), that constitute a transitional stage preceding that of mature soldier (Fig. 1a). In soil-feeding Termitinae, only the workers feed on organic matter and foster other members of the colony, which are unable to feed by themselves, by trophallaxis. Particularly, young larvae and white soldiers receive pure saliva, while mature soldiers receive regurgitated (stomodeal) food [36], as proctodeal trophallaxis was never reported in Termitidae [37,38].

Here we provide a detailed survey of *Wolbachia* infection in 15 termite colonies. Primary queens and kings, larvae, workers, white soldiers and mature soldiers were sampled in colonies of the very common soil-feeding termites *Cubitermes* spp. *affinis subarquatus* (Termitidae, Termitinae) that consist of four distinct phylogenetic species in the Lopé forest Reserve (Middle Ogooué, Gabon) [39]. DNA from individuals and pools of salivary glands were screened for the presence of *Wolbachia* using PCR assays targeting the *wsp* gene. *Wolbachia* sequences from *Cubitermes* spp. *affinis subarquatus* were previously obtained for *wsp* and four other genes: *ftsz*, *fbpA*, *coxA* and 16S rDNA [33]. Because experimental studies could not be conducted for soil-feeding termites owing to their complex ethology, ecology and life cycle, we modeled our data in order to explain the unexpectedly high rate of *Wolbachia* infection in some sterile adult stages such as white soldiers.

## Material and Methods

### Termite and tissue samples

Termites were sampled in 15 colonies of *Cubitermes* spp. A, B, C and D *affinis subarquatus* (Termitidae, Termitinae) in the Lopé forest Reserve (Middle Ogooué, Gabon) [39]. The authors are grateful to the authorities of Gabon represented by Mr E. Mamfoumbi of the ‘Ministère des Eaux et Forêts’ for permission to work in the Lopé Reserve. Termite samples were preserved in absolute ethanol at 4°C until DNA extraction. We tested 555 *Cubitermes* individuals for the presence of *Wolbachia*: 535 from neutral castes/stages (larvae, workers, white soldiers and mature soldiers) and all primary reproductives present at the time of the sampling i.e. 20 dealate queens and kings (Table 1). For DNA extraction of these samples, abdomens were removed to avoid contamination / PCR inhibition from gut content. Different tissues throughout the body were screened for two individuals per caste: head (without salivary glands), eggs, ovarian tissues and Malpighian tubules were dissected from queens, head and fat tissues from kings, head, thorax and legs from workers, white soldiers and mature soldiers. Two pools of salivary glands from ten workers were also dissected (Table 2).

**Table 1:** Prevalence of *Wolbachia* in 15 *Cubitermes* colonies and infection rates across different castes and stages.

Termite species	Colony	*Wolbachia*supergroup	AN *wsp*	infected queens	N_Q_	infected kings	N_K_	infected larvae	N_L_	infected workers	N_W_	inected white soldiers	N_WS_	infected mature soldiers	N_S_	N_tot infected_/N_total_ (%)
*Cubitermes* sp. A	T7	B	DQ124660	100%	1			83%	6	70%	10	50%	4	100%	6	21/27 (77.77%)
*Cubitermes* sp. A	T16	B	DQ124659	100%	1			87%	15	90%	10	100%	1	40%	10	28/37 (75.76%)
*Cubitermes* sp. A	T42	B	DQ124661	100%	1	100%	1	48%	21	40%	10	75%	4	80%	20	35/57 (61.40%)
*Cubitermes* sp. B	T5	A	DQ124665	100%	1	0%	1			0%	15	80%	5	0%	3	5/25 (20.00%)
*Cubitermes* sp. B	T26	A	DQ124667	100%	1					0%	9	75%	4	10%	10	5/24 (20.83%)
*Cubitermes* sp. B	T34	A	DQ124666	100%	1			68%	19	80%	15			87%	15	39/50 (78.00%)
*Cubitermes* sp. B	T37	A	DQ124663	100%	1	100%	1			0%	20	20%	5	7%	15	4/42 (9.52%)
*Cubitermes* sp. B	T38	A	DQ124664	100%	1	100%	1	21%	19	25%	12	100%	1	50%	14	17/48 (35.42%)
*Cubitermes* sp. C	T17	A	DQ124655	100%	1					88%	16	100%	1	0%	1	16/19 (84.21%)
*Cubitermes* sp. C	T24	A	DQ124654	100%	1	100%	1	64%	25	30%	10	100%	2	30%	10	26/49 (53.06%)
*Cubitermes* sp. C	T31	A *(wsp)*	FJ851084					90%	10	11%	9	70%	30	17%	6	32/55 (58.18%)
*Cubitermes* sp. C	T45	A *(wsp)*	FJ851085					70%	10	0%	3	100%	2	0%	5	9/20 (45.00%)
*Cubitermes* sp. C	T46	A	DQ124658	100%	1			56%	9	0%	10	67%	3	0%	10	8/33 (24.24%)
*Cubitermes* sp. C	TX	A	DQ124656	100%	1	0%	1	80%	10	10%	10	90%	10	0%	10	19/42 (45.24%)
*Cubitermes* sp. D	TD1	B	DQ124662	100%	1	100%	1			10%	10	100%	5	40%	10	12/27 (44.44%)
N total				13	13	5	7	90	144	55	169	57	77	56	145	276/555 (49.73%)
% infection				100%		71.43%		62.5%		32.54%		74.03%		38.62%		
								(54–71)		(25–40)		(64–84)		(31–47)		
								b		a		c		a		

*Wolbachia* supergroups were determined in Roy and Harry [33] for all colonies except T31 and T45. Colonies T31 and T45 were not submitted to a MLST analysis and only the *wsp* gene was sequenced. AN: Genbank Accession Numbers, N: number of individuals tested for each caste or stage. 95% confidence intervals are indicated in brackets. Tests for the significance of infection were performed via the Wald statistics using a generalized linear model (GLZ). Results obtained between castes are summarized using letters; infection rates that do not significantly differ share the same letter.

**Table 2:** Tissue distribution of *Wolbachia* in *Cubitermes* castes and stages.

Castes	Tissues	12S rRNA gene	*Wolbachia*
Queen (n = 2)	Head	+	+
	Eggs	+	+
	Ovarian tissues	+	+
	Malpighian tubes	+	+
King (n = 2)	Head	+	+
	Fat tissues	+	+
Workers (n = 2)	Head	+	+
	Thorax + legs	+	+
White soldiers (n = 2)	Head	+	+
	Thorax + legs	+	+
Mature soldiers (n = 2)	Head	+	-
	Thorax + legs	+	+
Pool of 10 workers (n = 2)	Salivary glands	+	+

Queens were from T5 (*C*. spA) and T42 (*C*. spB), kings were from T38 (*C*. spA) and T42 (*C*. spB), workers, white soldiers and mature soldiers were from T42 (*C*. spB) and workers’ salivary glands were from T34 (*C*. sp A) and T42 (*C*. spB). The occurrence of *Wolbachia* was tested using *wsp* PCR and recorded as positive (+) or negative (-). The DNA quality was tested by using 12S ribosomal RNA gene.

### DNA extraction

Total genomic DNA was individually isolated using Wilson buffer (Tris-hydrochloride 1M, EDTA 0.5M, sodium chloride 4.5M, sodium dodecylsulfate 20%, dithiothreitol, proteinase K), followed by a salting-out procedure. DNA from tissue samples and salivary glands was extracted using a 10% Chelex solution, which has been described as a means of increasing the signal from the PCR amplification of small amounts of DNA [40]. In order to discard *Wolbachia* false negatives, PCRs targeted on termite DNA were performed using forward primer SR-J-14233 (5′-AAG AGC GAC GGG CGA TGT GT-3′) and reverse primer SR-N-14588 (5′-AAA CTA GGA TTA GAT ACC CTA TTA T-3′) amplifying a fragment of 12S ribosomal RNA gene [41]. Low-quality DNA samples that did not amplify termite rDNA were rejected.

### PCR detection of *Wolbachia*

DNA from termites and tissues was screened for the presence of *Wolbachia* via Polymerase Chain Reaction (PCR) using *wsp* primers: wsp81F (5’-TGG TCC AAT AAG TGA TGA AGA AAC-3’) and wsp691R (5’-AAA AAT TAA ACG CTA CTC CA-3’) [42]. Each PCR reaction was performed in a total volume of 12.5μL, composed of 2X Taq PCR Master Mix (Qiagen, France), 0.5μM of each primer, 2.35mM MgCl2, 0.016mg/mL Bovin Serum Albumine and 2μL of template DNA. The conditions for amplification were initial denaturation at 95°C for 3 minutes then 35 cycles of (1) denaturation at 95°C for 30 seconds, (2) annealing at 45°C for 30 seconds, (3) extension at 72°C for 1.30 minutes and a final extension at 72°C for 10 minutes. A negative (sterile water) and a positive control (known infected queen DNA) were included in each PCR run. Each sample was tested twice and was considered to be infected when at least one of the PCRs was positive. Sequencing of *wsp* gene was realized for up to three sequences per colony and for salivary glands using BigDye Terminator Cycle Sequencing kit version 1.1 (Applied Biosystems). Sequences were obtained using an automatic DNA sequencer (AppliedBiosystems, ABI PRISM 310). Accession numbers are listed in Table 1.

### Statistical analyses

Infection data were analyzed using a generalized linear model (GLZ). Because the dependent variable (infection status) could be either 0 (absent) or 1 (present), a binomial error structure and a logistic link function were used. Colony and caste were considered as independent variables (categorical factors) and the interaction of both variables was added to build the logit model. The Wald test was used to determine the significance of the effects in the model. Binomial confidence intervals for the average infection rates of the different castes were calculated using a normal approximation.

A model has been built as follows, assuming that caste/stage changes are independent of *Wolbachia* status. Let *I(s*, *c)* being the rate of infection for the species *s* and caste *c* with species numbered from 1 to 4 and castes/stages from 1 (queen), 2 (larva), 3 (worker), 4 (white soldier) to 5 (mature soldier). Let *a(s*, *c)* the probability that an individual for the species *s* and caste *c* had lost infection between caste *c-1* and *c*. Let *b(s*, *c)* the probability that an uninfected individual at caste *c-1* becomes infected as being in caste *c*. Then the model of transmission rate between two castes can be expressed based on two different equations depending on the exact process for the gain of *Wolbachia*.

In the first case, the loss a(s,c) is applied to the frequency of infected I(s,c-1) individuals and the gain b(s,c) is applied to the non-infected (1-I(s,c-1)) individuals at the previous stage (c-1). The equation describing infection rate is therefore:

I(s,c)=1-a(s,c))⋅I(s,c-1)+b(s,c)⋅(1-I(s,c-1))I(s,c)=I(s,c-1)⋅(1-a(s,c)-b(s,c))+b(s,c) (1)

Alternatively, the gain could occur later during the current stage. The difference with the previous model is that the proportion of uncontaminated individuals to which the gain is applied is the proportion of the current stage (1-(*I*(*s*, *c*-1) (1-*a*(*s*,*c*)))) and not the one at the previous stage (1-(*I*(*s*,(*c*-1)) as in equation (1). Then the equation describing infection rate becomes:

I(s,c)=(1-a(s,c))⋅I(s,c-1)+b(s,c)⋅(1-((1-a(s,c))⋅I(s,c-1)))I(s,c)=I(s,c-1)⋅(1-a(s,c))⋅(1-b(s,c))+b(s,c) (2)

Briefly, in the first model, gain is applied to individuals not infected at the previous stage. In the second model, individuals can lose infection during the current stage and can also gain infection.

The parameters *a(s*, *c)* and *b(s*, *c)* have been fitted using maximum likelihood with binomial link. This general model can be simplified letting *a(s*, *c)* or *b(s*, *c)* independent of the species for *a(*., *c)* or *b(*., *c)*, independent of the caste for *a(s,.)* or *b(s,.)*, or independent of both the caste and the species for *a(.,.)* or *b(.,.)*. Furthermore, if *a(s*, *c) = 0*, no stochastic loss is allowed and if *b(s*, *c) = 0*, no gain is allowed.

For each of the 28 tested models (14 with equation 1 and 14 with equation 2), the quasi-likelihood of the observed rate at each stage 2 to 5 has been established and transformed to Akaike Information Content (AIC) [43]. AIC has been corrected for overdispersion (ĉ = 2.9) and small effective size using QAICc [44]. This is a ranking measure that takes into account the quality of the fit of the model to the data while penalizing for the number of parameters used.

The models with the lowest values of QAICc were retained as good candidate models and Δ_QAICc_ was calculated as the difference in value of QAICc between a particular model and the one with the lowest QAICc. Akaike weights (*w*_*i*_ = exp(-Δ_*QAIC*c_/2)) normalized to 1) were used to evaluate the relative support of various tested models [44]. Akaike weights can be directly interpreted as conditional probabilities for each model.

After a first round of selection, the selected model has been refined taking into account the fitted values of parameters. Parameters with overlapping confidence intervals were forced to be the same or null if they were not significantly different from 0. Standard errors on the parameter were estimated by inverting the Hessian matrix of second order derivatives of likelihood for each couple of parameters.

## Results

### Infection incidence in different colonies and castes

*Wolbachia* was detected in all 15 colonies. Infection tests showed that 50% (276/555) of individuals were *Wolbachia* positive with 90% (18/20) of the sampled queens and kings infected (Table 1). Global offspring infection rate was 48% (258/535) and per caste, 63% for larvae (90/144), 33% for workers (55/169), 74% for white soldiers (57/77) and 39% for mature soldiers (56/145). Mature soldiers and workers had a significantly lower mean infection rate than larvae and white soldiers (GLZ, p < 0.001). White soldiers had a significantly higher mean infection rate than larvae (GLZ, p = 0.0146).

Using a maximum likelihood method, two probabilities were estimated: the probability of an infected individual to become uninfected at the next stage and the probability of an uninfected individual to become infected at the next stage, using two different equations.

The best model has been selected using a two-step procedure. First the model describing the modality of gain has been selected (equation 1 or 2) and second the model has been simplified. The best model out of the 28 tested using Akaike weight (p = 0.78) includes a species effect for the stochastic loss of *Wolbachia* and a caste effect for the rate of gain described with the second equation (Table 3). The second selected model used also equation 2 (p = 0.11) and then the probability that the best model is described by equation 2 is around 0.95.

**Table 3:** Characteristics of the tested models. Selected model based on QAICc is shown in bold.

	Model	Loss	Gain	-Ln L	Parameters	QAICc	Akaike weight
1	1	a(.,.)	b(.,.)	390.21	1	209.12	0.0037
2	1	a(s,.)	b(.,.)	282.73	5	207.43	0.0086
3	1	a(s,.)	b(s,.)	275.11	8	212.66	0.0006
4	1	a(.,.)	b(s,.)	285.91	5	209.63	0.0029
5	1	a(., c)	b(.,.)	284.63	5	208.74	0.0045
6	1	a(., c)	b(., c)	275.37	8	212.84	0.0006
7	1	a(.,.)	b(., c)	279.66	5	205.32	0.0248
8	1	a(s, c)	b(s, c)	227.37	28	389.96	0.0000
9	1	a(., c)	b(s, c)	251.52	18	256.82	0.0000
10	1	a(s,.)	b(s, c)	249.77	18	255.62	0.0000
11	1	a(s, c)	b(., c)	236.95	18	246.79	0.0000
12	1	a(s, c)	b(s,.)	242.07	18	250.32	0.0000
13	1	a(s,.)	b(., c)	262.84	8	204.21	0.0433
14	1	a(., c)	b(s,.)	273.94	8	211.86	0.0009
15	2	a(.,.)	b(.,.)	298.05	2	209.76	0.0027
16	2	a(s,.)	b(.,.)	287.67	5	210.84	0.0016
17	2	a(s,.)	b(s,.)	285.17	8	219.60	0.0000
18	2	a(.,.)	b(s,.)	285.74	5	209.51	0.0031
19	2	a(., c)	b(.,.)	289.03	5	211.78	0.0010
20	2	a(., c)	b(., c)	274.78	8	212.44	0.0007
21	2	a(.,.)	b(., c)	275.17	5	202.23	0.1167
22	2	a(s, c)	b(s, c)	229.06	28	391.12	0.0000
23	2	a(., c)	b(s, c)	236.52	18	246.49	0.0000
24	2	a(s,.)	b(s, c)	234.59	18	245.17	0.0000
25	2	a(s, c)	b(., c)	237.00	18	246.83	0.0000
26	2	a(s, c)	b(s,.)	264.59	18	265.83	0.0000
**27**	**2**	**a(s,.)**	**b(., c)**	**254.43**	**8**	**198.42**	**0.7838**
28	2	a(., c)	b(s,.)	275.88	8	213.19	0.0005
29	Full			199.79	47		

Then, for the selected model (equation 2), the rate of gain for larva, worker and soldier castes was fitted or fixed to 0. Among the new models, the best selected model (p = 0.68) was for a species-specific rate of loss (ranging from 0.63 to 0.23 for species C and A respectively) with a null rate of gain for larvae, workers and soldiers and a fitted probability of 0.72, whatever the species, that a white soldier becomes newly contaminated during that stage (Fig. 1b). Although a constant loss of *Wolbachia* from stage to stage was detected (48% loss in average between two stages for the four species), a higher infection than expected (obtained when the probability of new gain was fixed to zero) was observed in white soldiers (mean %_obs_ = 74 ranging from 73% to 84% for species C and A, respectively, vs. mean %_exp_ = 17 ranging from 4% to 44% for species C and A, respectively if no gain was allowed) and was then responsible for a higher infection than expected in mature soldiers (mean %_obs_ = 38 ranging from 27% to 65% for species C and A vs. mean %_exp_ = 11 ranging from 2% to 34% for species C and A, respectively) (Fig. 1c).

### Infection incidence in tissues and salivary glands

*Wolbachia* was detected in eggs, ovarian tissues, Malpighian tubules, head and fat tissues of queens and kings, and in thorax + legs of workers and soldiers (Table 2). The heads of white soldiers and workers, and the two pools of salivary glands were also infected. Although very short, the *wsp* sequences from the salivary glands and those from the entire individuals showed 100% identity (318/318 bp).

## Discussion

### *Wolbachia* detection

*Wolbachia* detection was assessed by the way of PCR assays, a method which has been considered as the most efficient and easily applicable to large samples, such as in this study (N = 555) [45]. Here termite colonies were screened for the presence of *Wolbachia* using PCR assays targeting the *wsp* (*Wolbachia* surface protein) gene, which is 10 times more variable in its DNA sequence than 16S rRNA [46]. Despite the fact that the *wsp* gene was shown to be unreliable as a phylogenetic tool due to frequent recombination [47–49], it is still widely used for the detection of *Wolbachia* infection [50,51]. Particularly, the validity of *wsp* to diagnose *Wolbachia* A and B supergroups (i.e. those found in *Cubitermes* spp.) is well documented [45,52,53]. A negative point concerning the sensitivity of *wsp* gene was recently evoked by Simoes et al. [45] as *wsp* primers were shown to aspecifically detect DNA from bacterial genera that are phylogenetically close to *Wolbachia* such as *Rickettsia*, *Anaplasma* or *Ehrlichia*. In order to counter this problem, 1–3 *wsp* sequences per colony were obtained from different individuals and none of them showed aspecific amplification. Because nestmate sequences were identical to that of the queen, and because the gain is null in workers that are the sole stage to take food directly from the environment, transfer from infected humus was discarded and the current assumption is that the *Wolbachia* are inherited from the queen.

### Species pattern

All tested colonies were infected in a geographic range of about 0.1ha. As shown by Roy and Harry [33], *Cubitermes* spA and spD harbored B-supergroup *Wolbachia* while *Cubitermes* spB and spC harbored B-supergroup *Wolbachia*. The strict pattern “one *Wolbachia* strain/one *Cubitermes* species” suggests that interspecific horizontal transfers are not common. Compared to *Cubitermes* spB and spC hosting A *Wolbachia* supergroup (Fig. 1c), *Cubitermes* spA hosting B *Wolbachia* supergroup showed a particularly high level of infection for larvae (73%) (a stage which could give either future swarming females able to transmit the bacterium or sterile workers), and a lower rate of loss according to the model. Differences in transmission efficiency linked to the bacterial B-strain harbored by this species could be invoked. *Cubitermes* spD *affinis subarquatus*, hosting also B-*Wolbachia*, showed a moderate level of infection for larvae (40%) but is represented by a unique colony and rates are hardly interpretable in this way. To date, only one study conducted on *Aedes albopictus* demonstrated such a higher transmission efficiency of group B *Wolbachia* relative to supergroup A [54].

### *Wolbachia* loss in adult individuals

Results of the model indicate that rate of loss of *Wolbachia* between two successive stages is constant and independent on the species and caste/stages. Workers and mature soldiers showed a lower infection rate than larvae. This result is particularly clear in *Cubitermes* spC, where five on six colonies had sampled larvae and workers stages and a sharp decrease in observed infection rates. As shown by previous reports on flies, mosquitoes, planthoppers and ants, host age may influence *Wolbachia* infection levels and tissue tropism [12,26,30,55,56]. Particularly, infection data in ant colonies showed lower infection rates in adult workers compared to worker pupae [26,30]. It was suggested that selection did not favor heritable symbionts conservation in sterile castes (workers and soldiers) because infection could induce a decrease in colony productivity, and that symbiont curing from adult workers may be an adaptation that ensures efficient production of sexual females [2]. Hard environmental conditions were also proposed to explain the elimination of *Wolbachia* in foraging ant workers [26,57]. It is known that intense heat for short periods reduce *Wolbachia* densities and even cure some insects of their bacterial infections in laboratory environments [57–59] and these factors were proposed to have similar effects in natural insect populations [60].

Termite soldiers represent a true caste which is unique among social insects in function and development [61]. Dynamics of *Wolbachia* infection in the soldier caste were explored for the first time in this study and we showed that infection rate is higher in white soldiers than in mature soldiers. Here the white soldier-mature soldier difference is principally driven by *Cubitermes* spB, C and D since one colony in *Cubitermes* spA (T7) showed a reverse pattern.

### The case of white soldiers

In order to explain the higher infection rate in white soldiers, various hypotheses could be examined. Some of them can be discarded such as transfer from infected humus in white soldiers, because they do not feed by themselves, or the detection of *Wobachia* in alien white soldiers coming from colonies with a high rate of infection. However, colony fusion was never reported in *Cubitermes* species and if, would impact all stages and not only white soldiers. Although inquilinism is important in this genus [62] and particularly common in *Cubitermes* spp. *affinis subarquatus* in the Lopé Reserve (MH, personal observations), soldiers of inquiline genera other than *Cubitermes* are easily morphologically distinguished and no alien genotype was detected in other castes [39].

Another hypothesis could be a *Wolbachia* detection bias due to an increase of bacteria density in white soldiers compared to workers and mature soldiers. In the mosquito *Aedes albopictus* it was shown that the bacterium linked its own replication to that of its host cell, *Wolbachia* densities in eggs being greatest during embryonation and declining throughout diapause [63]. White soldiers are the site of multiple cell divisions: hard mandibles of the future soldier are constructed, salivary glands change their shape and many other morphological modifications occur [36]. White soldiers could constitute a preferential stage for *Wolbachia* replication, thus facilitating the detection of infections. In order to explore this hypothesis, sensitivity tests were carried out using serial dilutions of a number of infected samples from 1 down to 1:100 and these tests showed positive amplification of *wsp* gene, indicating that molecular tools used to detect infection are very powerful. Experiments using real-time quantitative PCR to compare *Wolbachia* density in white soldiers and workers could complement these tests.

The 28 tested models (14 for equation 1 and 14 for equation 2) indicated that *Wolbachia* transfer via horizontal routes between members of the colony (equation 2) has 95% of chance to be the best models among those tested to explain the observed pattern of infection. Among the 14 models, the ones with only stochastic loss of *Wolbachia* and no gain are not supported at all (p<0.001). It was found that *Wolbachia* occurred in reproductive organs of queens and kings but also in somatic tissues of all individuals (head, thorax and legs). The broad tissue tropism of *Wolbachia* is known in many arthropod species [11–13,27,64]. The high rates of infection in white soldiers, associated with the detection of *Wolbachia* in salivary glands of workers, could be the result of a recontamination by infected saliva via trophallaxis between feeding workers and white soldiers. An experimental study concerning trophallaxis and feeding in *Cubitermes fungifaber* demonstrated that white soldiers received salivary secretions, while mature soldiers received regurgitated food (stomodeal) from workers [65]. Field observations also showed that Termitidae workers fed all the colony members but that only white soldiers and young larvae were heavily loaded with pure saliva [36,66].

Various experimental studies described horizontal transmission of *Wolbachia* during close contact of individuals. Rigaud and Juchault [67] reported direct transfer of the symbiont in the isopod *Armadillum vulgare* via blood-to-blood contacts, probably during moulting or by the way of predator injuries. More recently, Le Clec’h et al. [68] proposed cannibalism and predation as paths for horizontal passage of *Wolbachia* between isopods. Direct *Wolbachia* transmission between *Trichogramma* wasp larvae sharing a same food source has also been described when superparasitism occurred, probably by consumption of infected larvae by uninfected ones or during blood-to-blood contact [14,15]. *Wolbachia* has been detected in the salivary glands of several insects, such as mosquitoes [69] and insects feeding on host plants [70,71]. In these last examples, saliva was suggested to be a medium for microorganism transmission, including *Wolbachia*, in an insect-host plant-insect pathway.

In termites, recurrent transfer via feeding workers may be of functional importance and produce other effects than those currently known for *Wolbachia*. In *Acromyrmex* ants, it has been suggested that the high intensity of *Wolbachia* found in the haemolymph and faeces could enhance transmission via parasitoid and the faecal-oral route, or have a role in modulating the immune response of the host [72]. Similarly, it has been proposed that *Wolbachia* found extracellularly in the gut may have a nutritional role [73]. Here the high proportion of infected white soldiers could represent a side-effect of the specialization of *Wolbachia* in the alimentary canal, due to the same “trophically dependent” behavior of white soldiers and larvae (comprising larvae destined to develop into sexual individuals). The vertical transmission would be then supplemented with horizontal transfers via trophallactic exchanges between infected individuals and future imagoes. Examining the prevalence of infection in the reproductive lineage (nymphs and alates) would probably provide useful information concerning the transmission patterns and dynamics of *Wolbachia* infections. These results suggest new ways of transmission to explore in other social insects and more generally in arthropods since such close interactions between individuals are not limited to termites: trophallaxy and brood care that are common in social insects also occur in burying beetles and earwigs, where larvae or nymphs are fed with regurgitations.

A set of explanations implying patterns of caste differentiation has to be mentioned but cannot be tested with current data because of lack of degree of freedom in the models (more parameters to estimate than information) or lack of information (*i*.*e*. absolute age of individuals). In *Cubitermes* species, only a small subset of workers is destined to transition to white soldiers as soldiers represent a small proportion of individuals relative to workers (1.6% in *C*. *fungifaber*, [74], 0.8–5.1% in *C*. *sankurensis* [75], 1.4–8.4% in *C*. *subcrenulatus* [76]). Regarding to the terminology of Oster and Wilson [77], these species with a minimal investment in defence are classified as tychophile species [78]. *Cubitermes* soldiers differentiate from the molt of workers that are not morphologically different from other workers [74] and there is no obvious way of knowing whether an individual is destined to become a soldier or not.

In all models, caste/stage changes are assumed to be independent of *Wolbachia* status. However, it remains possible that white soldiers have elevated levels of infection compared to workers because workers infected with *Wolbachia* preferentially develop into white soldiers. Similarly, lower infection rates detected in workers compared to larvae could be explained by the fact that the subset of the larvae transforming into nymphs (i.e. future swarming alates), and not in workers, would be preferentially infected by *Wolbachia*. This raises interesting questions about the benefits and costs of *Wolbachia* in different members of a colony.

White soldiers could also show elevated levels of infection compared to workers because old workers, which would be more likely to have lost *Wolbachia* if *Wolbachia* decline is constant over time, outnumber younger white soldiers (Fig. 1). Our model can appear quite simplified because *Wolbachia* loss and/or gain are treated stage per stage rather than a timely process (e.g. workers are treated as a stage preceding white soldiers). Although very interesting, it seems nevertheless difficult to test or model these hypotheses because individuals could not be traced for infection and life trajectory.

If caste/stage changes are independent of *Wolbachia* status, the more reliable explanation to the high infection rate observed in white soldiers is that *Wolbachia* transfer could be a general phenomenon involving direct transmission from individual to individual via saliva during trophallactic exchanges in termites.
